# Application of PVC pipes as an adjustable bilateral traction device in lower limb fractures

**DOI:** 10.1186/s12891-023-06847-3

**Published:** 2023-09-14

**Authors:** Hongshuo Sun, Peng Li, Gangqiang Du, Jianhao Jiang, Kaikai Song, Hongzhi Liu, Xinjun Zhang, Long Jia, Kai Zhang, Shuye Yang, Zhigang Wang

**Affiliations:** Department of Orthopaedic Trauma, Binzhou Medical University Hospital, No.661 Huanghe 2Nd Road, Binzhou, Shandong 256603 People’s Republic of China

**Keywords:** Bilateral distraction, Lower extremity fracture, Bone traction, Closed reduction

## Abstract

**Objective:**

To introduce a new type of simple adjustable bilateral bidirectional polyvinyl chloride (PVC) tube traction device and discuss the value of using this device before surgery in patients with lower limb fractures.

**Methods:**

To introduce the manufacturing process of an adjustable bilateral traction device made of PVC pipes. From August 2018 to November 2019, the data of 36 patients with lower limb fractures who were treated with this traction device were retrospectively analysed. The treatment outcomes were analysed, including length of both lower limbs, fracture reduction, lower limb mobility, visual analogue scale (VAS) score, incidence of complications, and patient satisfaction.

**Results:**

All patients were able to move the affected limb immediately after using the device. The patient's pain was significantly reduced, they were able to turn over freely during bed rest, and the length of the affected limb was restored to that of the healthy limb. Thirty-four (94.5%) patients were satisfied with the reduction of the fracture end, 2 (5.5%) patients with tibiofibular fractures showed angular displacement of the fractured end and satisfactory reduction after the position of the bone traction needle was adjusted; 7 (19.5%) patients developed deep vein thrombosis of the affected lower limb during traction; there was no decubitus or vascular nerve injury, and the overall complication rate was 25% (9/36). All the patients and their families were satisfied with the results of this treatment.

**Conclusion:**

The aim of this study is to introduce a new type of traction device. It is advantageous in that it is light weight, low cost, easy to assemble, promotes immediate movement of the affected limb after assembly, improves patient comfort and can be used with a titanium steel needle for MRI examination under traction. In the clinical setting, it has been shown to be suitable for the temporary treatment of patients with lower leg fractures prior to surgery, particularly patients who, for various reasons, require nonsurgical treatment in the short term.

**Supplementary Information:**

The online version contains supplementary material available at 10.1186/s12891-023-06847-3.

## Highlights

In this work, we evaluated a new type of bilateral traction device with PVC tubes, it is suitable for patients with lower leg fractures before operation temporary treatment, especially patients with lower limb fractures who are in intensive care and neurosurgery units MRI examination is required while receiving treatment. This device is lightweight, inexpensive and easy to assemble. Its greatest advantage is that the affected limb can be moved immediately after installation, so the limb is no longer limited to the traction bed. It is also convenient for nursing and improves the comfort of patients. In addition, the device can be used with titanium steel needle for MRI inspection under traction.

**Supplementary Information:**

The online version contains supplementary material available at 10.1186/s12891-023-06847-3.

## Introduction

Bone traction is commonly used in trauma orthopaedics, as it can provide both reduction and fixation [1]. In the clinic, some patients cannot undergo immediate fracture surgery due to contraindications to surgery or severe head, chest or abdominal injuries, so lower limb bone traction is usually performed first in these patients [2]. MRI is increasingly being used in orthopaedics because of its high sensitivity in accurately diagnosing soft tissue injuries, especially in cases of multiple injuries throughout the body caused by high-energy trauma. Moreover, high-energy trauma injuries, such as lower limb fractures combined with head injuries, often require multiple MRI scans to assess the damage and effectiveness of treatment. Lower limb fractures combined with spinal fractures often require MRI scans to assess nerve damage, and knee injuries often require MRI scans to identify damage to the meniscus, articular cartilage and ligaments of the knee. Currently, most bilateral reverse traction devices used in clinical practice consist of a bone traction needle and a steel-structure external fixator, which is heavy and cumbersome and therefore prevent patients from effectively performing lower limb activities. In addition, the steel outer frame cannot withstand MRI examination its ability to block X-rays, thereby potentially hindering the observation of fracture end reduction during re-examination. Moreover, the cost of such examinations with this device is a significant economic burden for patients in primary hospitals. To address the above disadvantages in clinical practice, provide a solution to its limited use in primary care facilities, and reduce the economic burden on patients, we designed a bilateral bidirectional traction device with an adjustable PVC tube for lower extremity fractures.

## Materials and methods

From August 2018 to November 2019, 36 patients with lower limb fractures were temporarily treated with an adjustable PVC tube double-counter traction device during hospitalisation in our department. These are patients who require prolonged traction. This device was chosen to improve comfort, facilitate nursing care, facilitate MRI scans, etc. In this study, we retrospectively analysed the relevant data of these patients, including demographic data (age, sex, mechanism of injury), fracture site, fracture type, concomitant injuries, degree of limb shortening, application time, traction time, complication rate and patient satisfaction. The study was approved by the ethics committee of Binzhou Medical University Hospital (No. 2018–015-01). All procedures were performed in accordance with relevant guidelines and regulations. Informed consent was obtained from all individual participants included in the study.

*Inclusion criteria were as follows:* (1) patients who could not undergo surgery for lower extremity fractures in time; (2) patients with lower extremity fractures who had underlying disease such as acute cerebral infarction, acute myocardial infarction and liver and kidney failure that made them unsuitable for surgery; (3) patients who had undergone preoperative temporary treatment with the adjustable PVC tube bilateral bidirectional traction device; and (4) patients who had performed preoperative self-care and had good limb function***.***

*The exclusion criteria were as follows:* (1) combined calcaneus or knee fractures not amenable to traction; (2) bone tumours, diabetes, and chronic cardiovascular and cerebrovascular disease; (3) inability to tolerate local infiltration anaesthesia; (4) poor compliance or inability to undergo treatment; and (5) incomplete clinical and imaging data.

### Materials

**Fig. 1:**
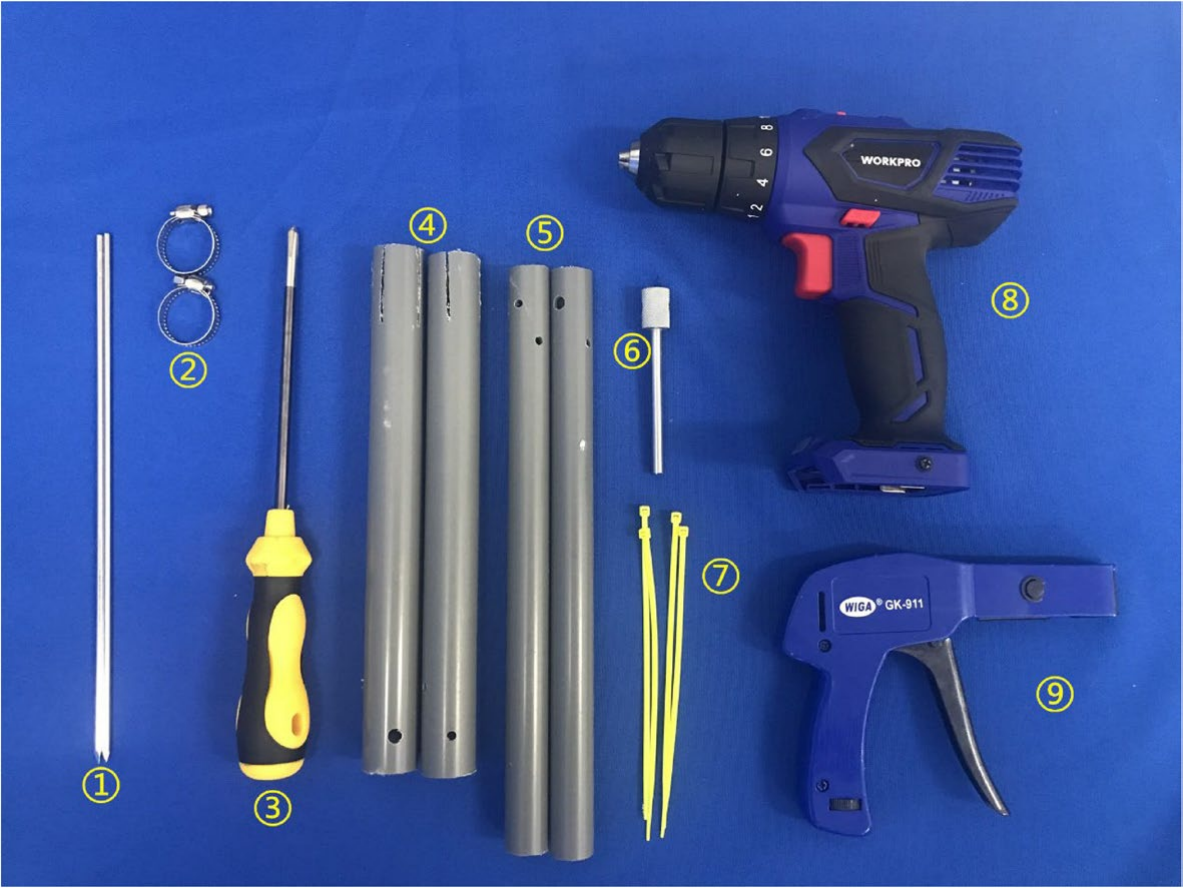
The materials and tools required to make the adjustable PVC tube double reverse traction device. ①Bone traction pin (3.5 * 250 mm) ②Stainless steel hoop ③Screwdriver ④PVC thick tube ⑤PVC thin tube ⑥Custom-made bone traction pin bending tool ⑦Self-locking nylon tie (3.5 *150 mm) ⑧Electric drill ⑨Nylon tie tightener

The materials used for the adjustable PVC tube double reverse traction device included PVC tubes, bone traction needles, self-locking nylon ties and stainless steel neck rings (Fig. 1). The sterile bone traction needle is a steel or titanium needle commonly used in clinical practice, size 3.5*250 mm. All other materials are commercially available and can be sterilised by low temperature plasma prior to use. PVC tubes are available in two sizes: 25 mm in diameter (thick tubes) and 20 mm in diameter (thin tubes), both with a wall thickness of 2 mm.

#### Traction operation

The procedures described in this report were performed by the same team of orthopaedic trauma surgeons on the ward, and all patients were operated on under local infiltration anaesthesia in the supine position. Different types of traction were chosen to maximise the range of motion near the fractured end of the joint, depending on the type of fracture and surgical approach.

#### Assembly of the PVC tube

**Fig. 2:**
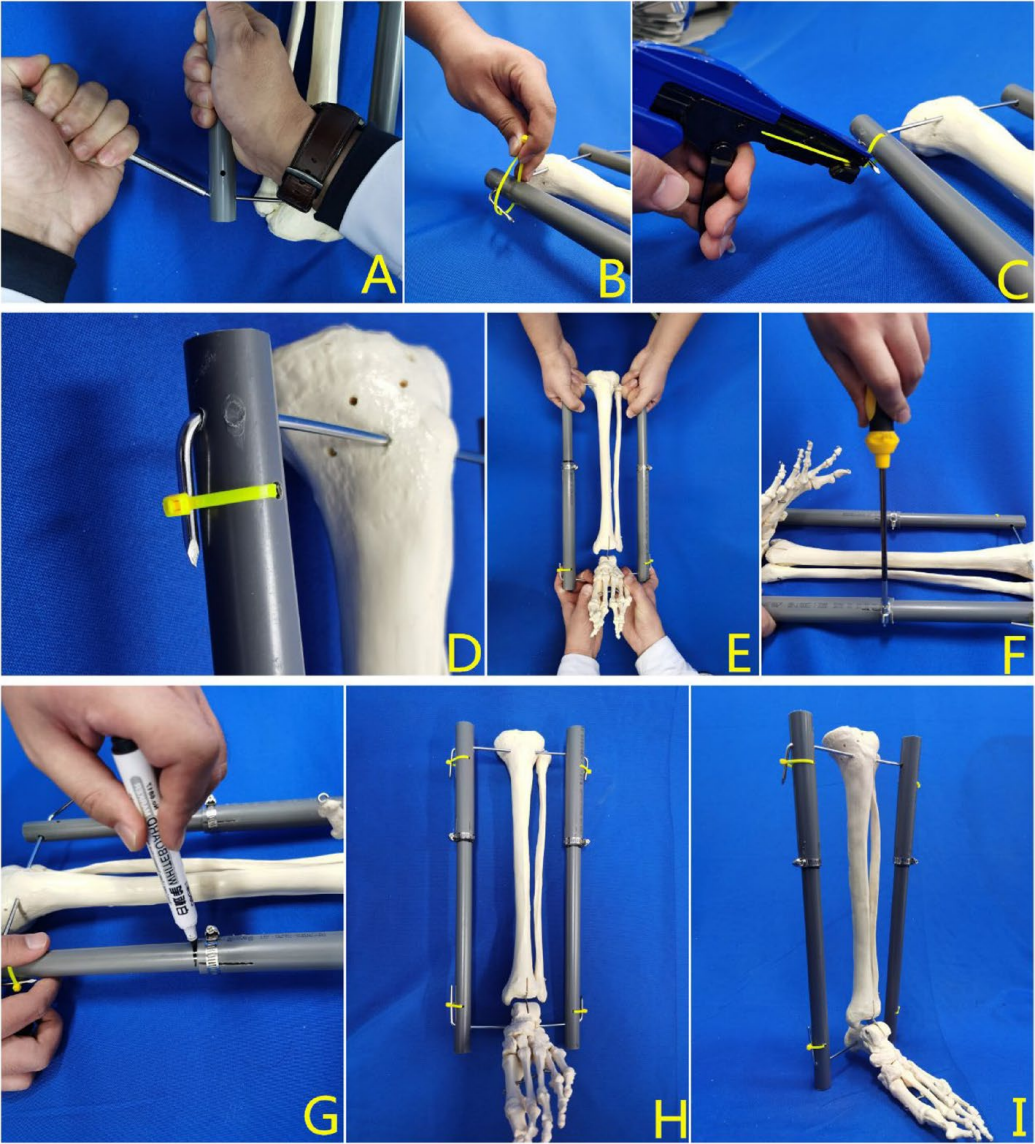
The assembly process of the adjustable PVC tube double reverse traction device. **A** The custom-made bending tool was used to bend the lateral bone traction needle of the official cavity. **B-D** The self-locking nylon tie was passed through the 4.0 mm channel in the anterior and posterior direction, and was firmly combined with the PVC tube and the bone traction needle by using a tensioner. **E** The assistant held the affected limb. **F** After the bone traction needle was obviously bent, the steel hoop was tightened. **G** A marker was used to mark the surface of the thin tube at the end of the thick tube. **H-I** The finished drawing of the device

A suitable set of PVC tubes was selected according to the length of the affected limb: length of the thick tube = distance between the two traction needles (L)*1/3 + 3 cm; length of the thin tube = distance between the two traction needles (L)*2/3 + 7 cm + 3 cm. The length of the thin tube embedded in the thick tube was 7 cm, which has been shown to be sufficient to maintain the stability of the device in the clinical setting. The length of the PVC tube outside the ends of the bone distraction pins was 3 cm, and it was designed for patients with fractures and open injuries to the posterior aspect of the leg to prevent contact between the affected limb and the bed. The thick and thin tubes were then integrated into a single structure, and the longitudinally cut end of the thick tube was inserted into the steel ring. For the two holes with different horizontal diameters at both ends of the PVC tube, the side with the 8.0 mm hole was placed close to the skin, and the 4.0 mm hole was placed on the opposite side to allow the bone traction needle to pass through the tube and bend. After the bone traction needle had penetrated each canal, a custom-made bending device was used to bend the portion of the bone traction needle reserved on the outside of the PVC tube, with the distal and proximal tips of the bone traction needle tips pointing in opposite directions. During bending, it was necessary to support the opposite bone traction needle with one hand to increase the counterforce. The self-locking nylon tie (3.5 *150 mm) was passed through the 4.0 mm channel in an anteroposterior direction, the needle tip was bent along the tube wall, and the tie was secured with the tie retractor to prevent the PVC tube from moving along the bone traction needle to the limb side. The assistant and the patient's family members were instructed to hold the bone traction needles at both ends and pull them in opposite directions. When the main body of the bone traction needle was bent, the steel ring was locked with a screwdriver. Based on clinical experience, the traction force at this point is approximately 4–8 kg. The steel ring was also designed to be directed away from the fractured end of the fracture prior to manipulation, and the presence of retraction at the fracture end could be observed in real time by marking the area on the thin tube next to the thick tube with a pen (Fig. 2). The time taken to complete the procedure was then recorded.

#### Posttraction treatment

The lengths of both lower limbs were measured immediately after the completion of traction to determine whether they were consistent. On the second day after traction, an imaging study was performed with radiographs of the two adjacent joints of the fractured end of the fracture to allow timely adjustment of the device to increase the opposing traction forces of the two bone traction pins. A 75% ethanol solution was used to disinfect the needle track twice daily, and the area around the needle track was kept clean and dry. Changes in skin and soft tissue sensation and blood circulation were monitored, and symptomatic treatment was given in a timely manner. On the day before the surgery, the degree to which the patient could lift the affected limb with a straight leg and the occurrence of complications were measured and recorded, and surgical treatment was performed after exclusion of patients with obvious surgical contraindications.

## Results

**Fig. 3:**
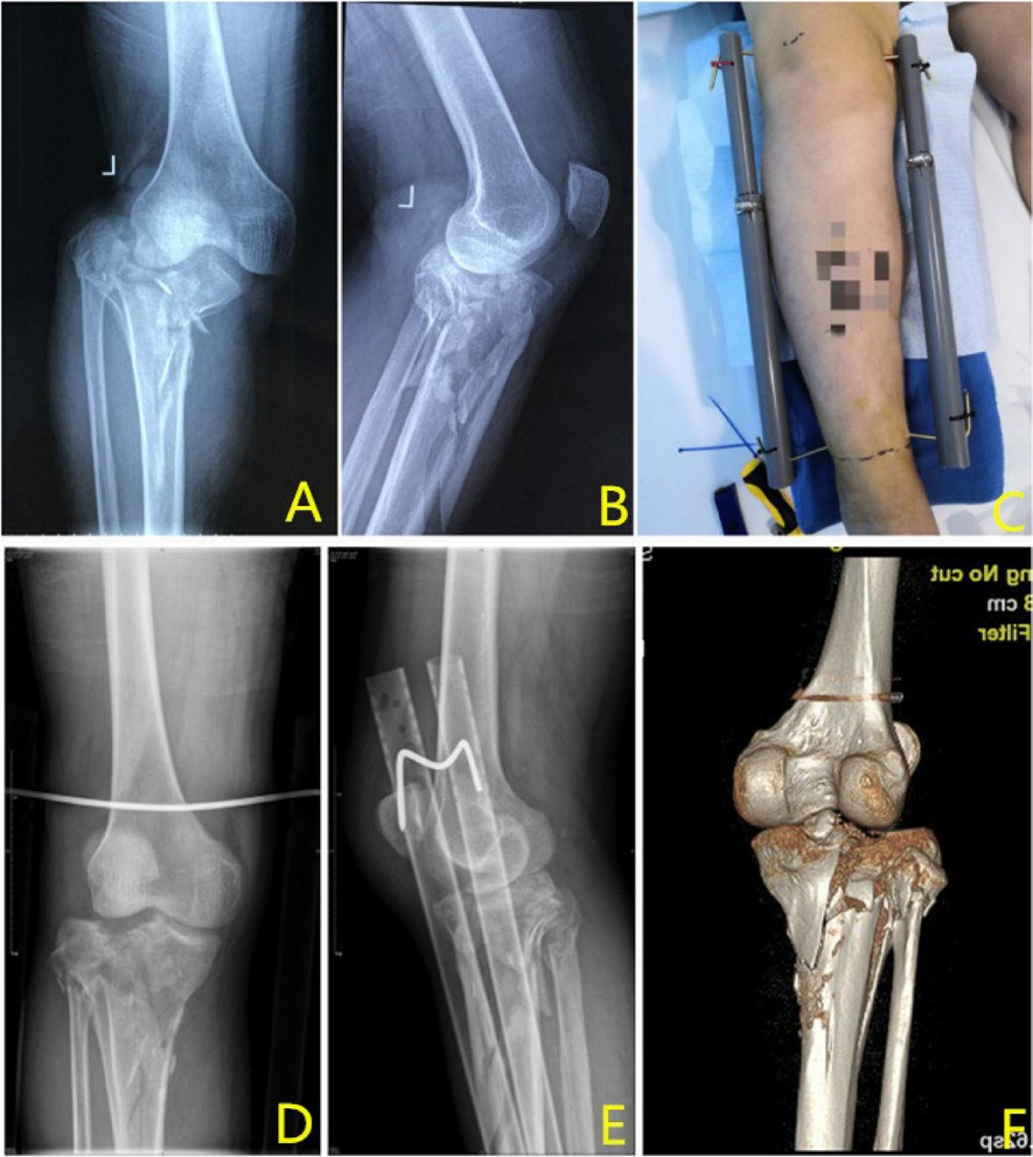
Traction effect on a tibial plateau fracture

**Fig. 4:**
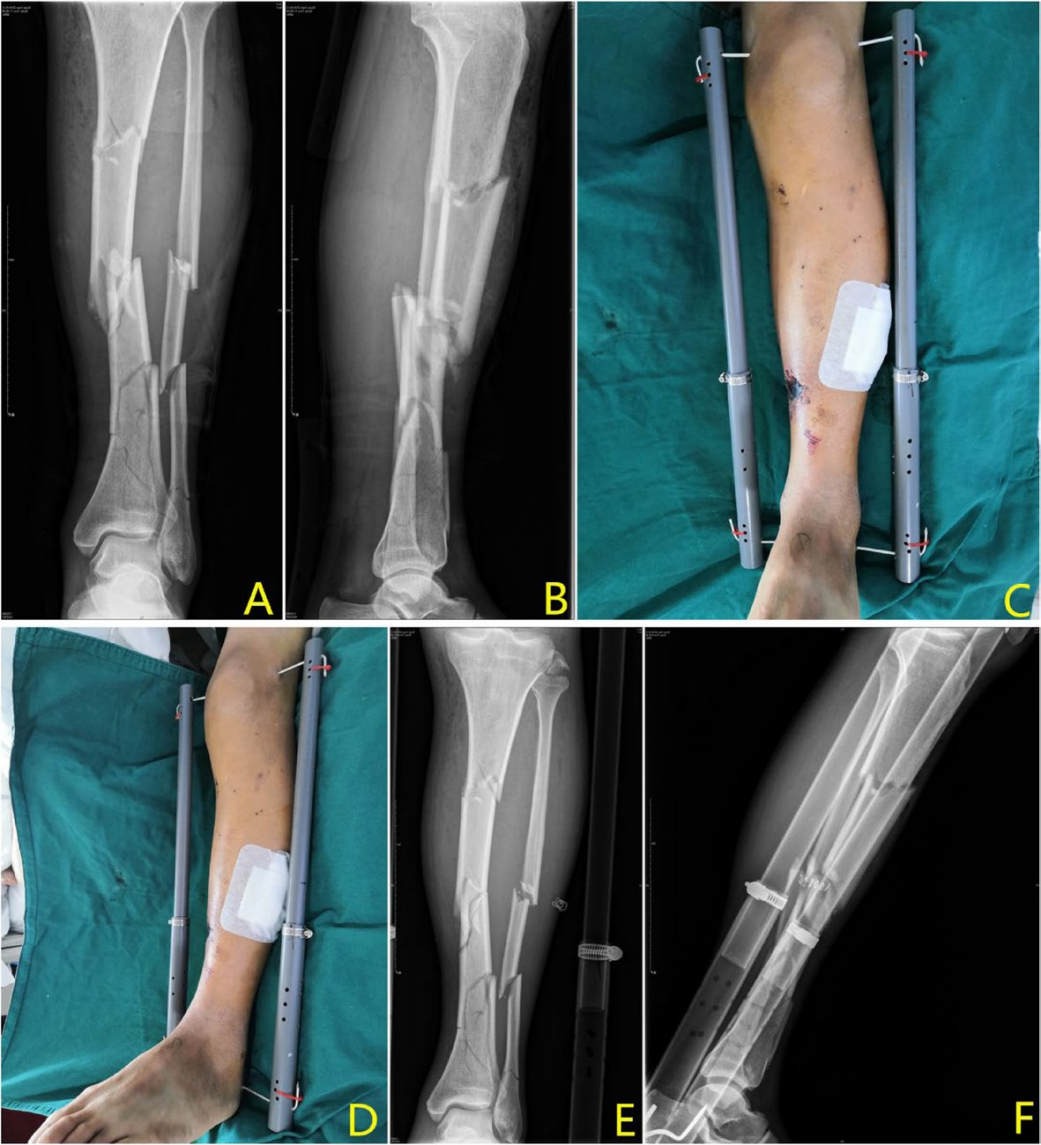
Traction effect on a tibiofibular fracture

**Fig. 5:**
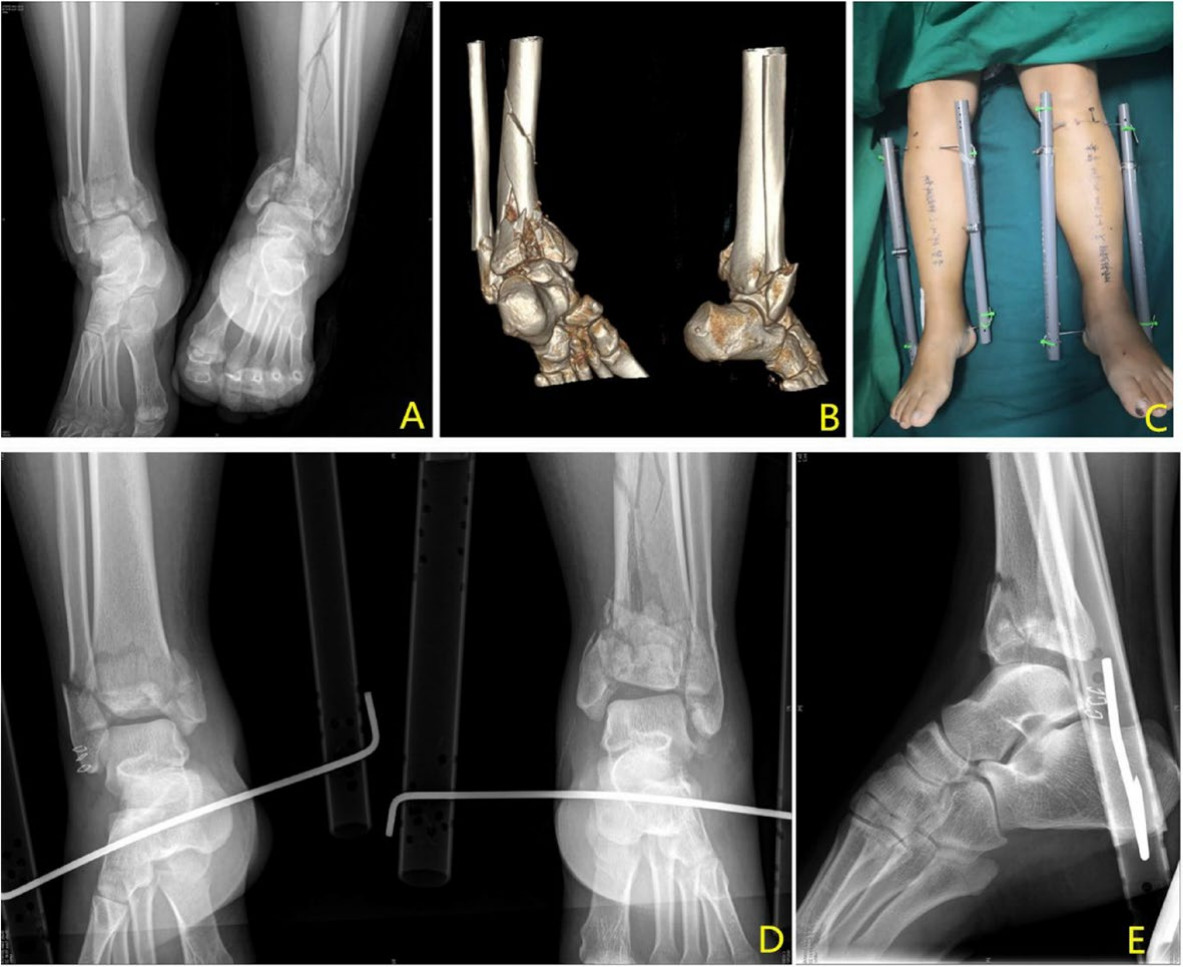
Traction effect on a Pilon fracture

A total of 36 patients (23 males and 13 females) were included in this study, with a mean age of 47.28 ± 16.96 years. There were 9 tibial plateau fractures (AO41B/C: 2/7), 3 pilon fractures (AO43C), and 24 tibiofibular fractures (AO42A/B/C: 5/11/8), including 17 fractures in the upper two-thirds of the region and 7 fractures in the lower one-third. Twenty-two (61%) patients had sustained fractures after car accidents. Only 6 (16%) patients developed an injury after a fall, 4 (11%) patients fell from a height, and 4 (11%) patients were hit by a heavy object (Table 1). The mean operating time was 16.92 ± 2.26 min. All patients received final treatment when they were well enough, and the mean traction time was 12.39 ± 7.22 days (Table 2). All patients in this study tolerated local infiltration anaesthesia without adverse effects, expressed their acceptance of the use of this device before discharge and were satisfied with the treatment results (Figs. 3, 4 and 5).

**Table 1:** Demographic data of the patients

Demographic data		Number of patients
**Age (years)**	Range 13–76 mean 47.28 ± 16.96	36
**Sex**	Male	23
	Female	13
**Type of fracture**	Tibial plateau fracture	9
	Middle and upper tibiofibular fracture	17
	Distal tibiofibular fracture	7
	Pilon fracture	3
**AO classification**	AO41B	2
	AO41C	7
	AO42A	5
	AO42B	11
	AO42C	8
	AO43C	3
**Mechanism of injury**	Traffic accident injury	22
	Fall from height	4
	Fall while walking	6
	Hit by heavy objects	4
**Shortening of the limbs(cm)**	Range 0.5–3.7 mean 2.18 ± 0.87	36

**Table 2:** Shows the traction details

**Traction details**		**Range**	**Mean**	**Number of patients**
**Surgery duration (min)**		13–23	16.92 ± 2.26	
**Traction duration(d)**		1–33	12.39 ± 7.22	
**VAS score**		3–7	4.92 ± 1.11	
**Limb mobility(°)**		10–60	40.53 ± 13.09	
**Reduction**	Effective			31
	Ineffective			5
**Complication**	DVT			7
	Displacement			2

Twenty (55%) patients had combined fractures or injuries elsewhere. Eleven (30%) patients with small areas of lower limb skin injury on admission underwent debridement and suturing in the ward, and the wounds healed with standard postoperative dressing changes. Three (8%) patients with large areas of soft tissue injury and varying degrees of bone exposure underwent debridement and suturing with VSD Biofilm Cover in the emergency operating room. Double reverse traction was also applied in the operating theatre. None of the patients had lower limb nerve dysfunction before traction or lower limb nerve injury after traction, and all patients had good blood supply.

Shortening of the affected limb occurred in 16 (44%) of 36 patients before traction. The magnitude of shortening ranged from 0.5–3.7 cm, with a mean of 2.18 ± 0.87 cm, and all limbs returned to the normal length after effective traction. All patients underwent lower extremity X-ray examinations on the 2nd day after traction, and the results showed that the fracture ends were completely retracted without intercalation in 31 (86%) patients. In 5 (14%) patients, the fracture end was partially retracted but completely retracted after adjustment of the traction force.

The VAS score after traction was 0–3 points in 4 patients, 4–6 points in 30 patients, and 7–10 points in 2 patients, with an average score of 4.92 ± 1.11 points. Patients with a score of more than 7 points complained of pain that interfered with sleep, and the pain disappeared after treatment with a reduced strength of traction and pain medication.

After traction, the patients could actively elevate the affected limb, had a degree of adjacent joint mobility within the normal range, and could actively straighten and elevate the leg 40.53 ± 13. 09° one day before surgery. Patients were able to roll over, sit up spontaneously and even walk without weight on the affected limb. Two patients with multiple traumas in the intensive care unit and three patients with traumatic brain injuries had limited function of the affected limb after traction.

No pulmonary embolism or cerebral embolism occurred, but seven (19.5%) patients experienced deep vein thrombosis on the affected side during traction. Thrombosis was stably controlled in 5 patients after oral administration of 15 mg rivaroxaban and in 2 patients after implantation of an inferior vena cava filter. In two patients with fractures of the lower third of the tibia and fibula, there was anterior angular displacement of the fracture end 1 and 2 days after distraction, respectively, and the length of the affected limb returned to the normal length after the position of the bone distraction pin was adjusted for redistraction. None of the patients had bed rest complications, such as decubitus injury or pneumonia.

## Discussion

Due to rapid economic development, the transport industry is becoming increasingly advanced, and the incidence of lower leg fractures is increasing [3]. The types of fractures caused by traffic injuries are mostly direct and violent and are often associated with soft tissue swelling, open fractures, and a combination of severe multiple head, chest and abdominal injuries [4]. Patients with the above conditions or in poor general health may not be candidates for immediate surgical treatment. Effective preoperative traction can not only relieve patient pain but can also prepare the limb for future minimally invasive closed fracture reduction [5, 6].

Traction is the use of external, opposing forces on the limb to treat injuries, and it can be considered an adjuvant therapy [7]. Bone traction is a long-established and effective treatment method used in trauma orthopaedics that involves inserting the bone traction needle into a relatively rigid position of the bone and pulling against the external force to displace the fold to achieve and maintain reduction [8]. Codivilla made an important contribution to the development of bone distraction by treating lower limb fractures with calcaneal distraction in 1903 [9]. Subsequently, Herzberg invented the distraction bow, which led to the further development of bone distraction [10]. Kirschner modified the traction pin and invented the K-wire, which was sufficiently strong to pass through the bone and maintain the traction force, and bone traction was finally widely popularised and used [11].

Traditional bone traction, including calcaneal tuberosity traction, tibial tuberosity traction and femoral supracondylar traction, is widely used in clinical practice due to its advantages including its ability to provide a large traction force, long traction duration and effective adjustment of the limb, and its important role in the treatment of lower limb fractures [12]. However, the above traction methods are unidirectional traction methods, which cannot correct the lateral displacement of the fracture end. Additionally, with these methods, the patient has difficulty turning over while lying in bed, the tractioned limb is confined to the traction frame, and the patient is prone to complications such as pressure ulcers, lung infection, and deep venous thrombosis of the lower extremities [13]. By combining the theories of traditional traction technology, Professor Zhang proposed the concept of "homeopathic reduction" and invented an intraoperative double-reverse traction rapid reduction device for the treatment of tibial plateau fractures. A number of improvements have been made, and this device can be used for almost all types of lower limb fractures [14–16]. With the clinical application and improvement of this device, the bilateral reverse traction device has been widely used in patients with multiple severe leg fractures that require elective surgery.

However, there are still some drawbacks of the preoperative bilateral reverse traction devices used in clinical practice. For example, 1. The main structure is mostly made of steel material, which is large and seriously interferes with the patient's ability to perform lower limb activities; 2. It has a high price, which increases the cost during hospitalisation; 3. Patients using this device cannot undergo MRI examination. Therefore, we decided to develop a new traction device for patients with lower leg fractures.

**Fig. 6:**
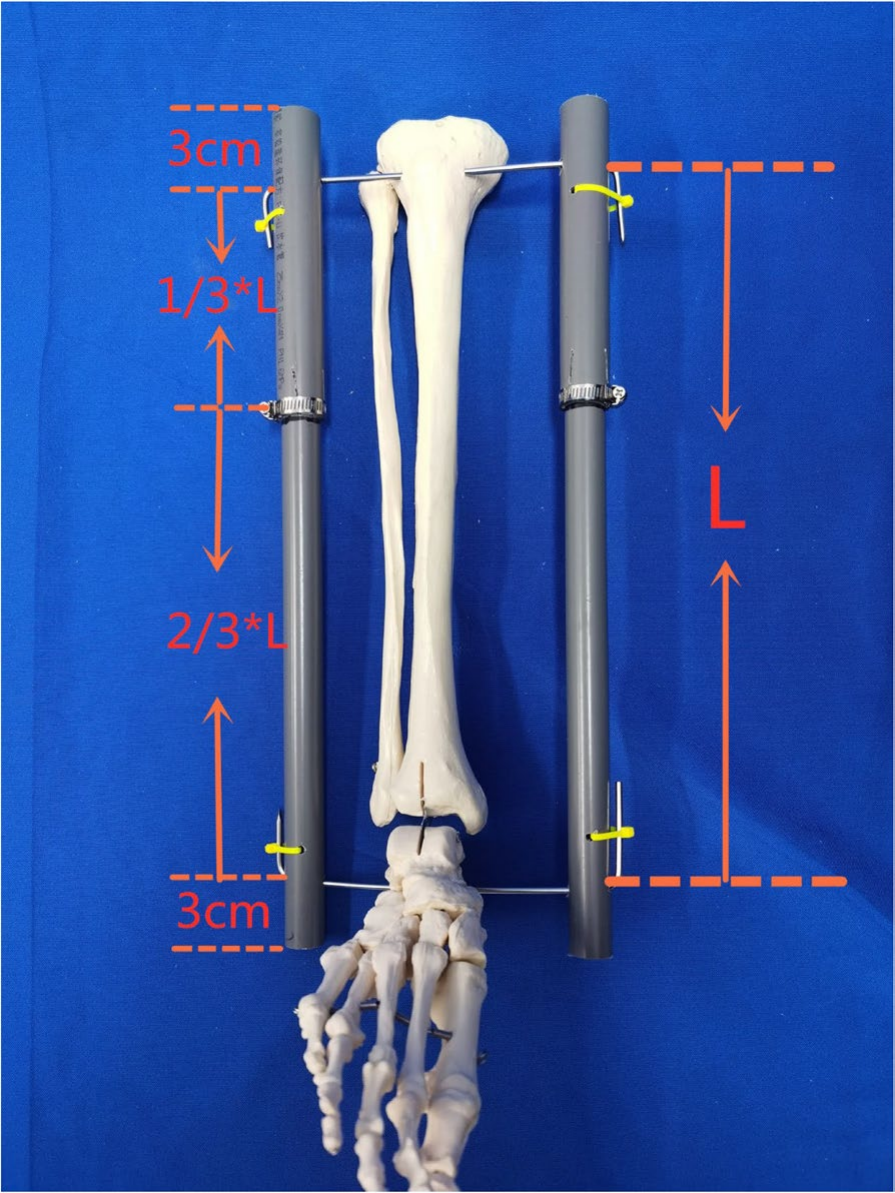
Model of the adjustable PVC tube double reverse traction device

The main structure of the device consists mainly of two bone pulling pins and four PVC tubes (Fig. 6). PVC tubes are derived from water pipes commonly used in homes and have a hard texture, strong bending resistance, and low price. The installation time of this device is short, and it can be quickly applied to fracture patients. The average installation time is approximately 16.92 ± 2.26 min. The position of the fracture end can be determined by X-ray after the patient has been injured. When the device is constructed, the contact points of the two PVC tubes are placed far away from the stainless-steel hoop, which is convenient for imaging scans after traction. Depending on the analysis of the patient's injury after admission, we can choose different materials for the bone traction needle. If we suspect that the patient has ligament or tendon injuries or other soft tissue injuries that require MRI examination, we prefer a titanium steel needle for traction.

The self-locking nylon ties at the four corners of the traction device with the bent bone traction needle can effectively prevent the PVC tube from sliding towards the limbs, which also solves the anti-rotation problem of the device and enables the bone traction needle and PVC tube to form a firm whole structure. We used an electric drill to drill 8.0 mm and 4.0 mm holes on the inside and outside of both ends of the PVC tube, respectively. The hole with the larger diameter is made closer to the skin, which increases the angle of needle penetration and significantly reduces the time required for installation. Moreover, the diameter of the lateral hole matches that of the bone traction needle, which is also beneficial for bending the bone traction needle. The lengths of both lower limbs were measured in the imaging results immediately after traction, and the device was adjusted appropriately, according to the results of the affected limb, to improve traction efficiency.

The cause of pain after fracture is mainly a series of reactions produced by the nerve endings of the periosteum, which are stretched and fed back to the cerebral cortex [17]. In our study, we found that the pain of patients treated with bilateral bidirectional traction using adjustable PVC tubes was significantly relieved compared with the pain before traction.

With the exception of some patients with multiple injuries and traumatic brain injuries, the patients showed significantly improved lower limb elevation results and adjacent joint mobility after traction, and the patients were able to move freely on the bed or even walk without weight on the affected limb, further confirming the effectiveness of this device in terms of traction strength. Additionally, the device is lightweight, so patients can effectively perform activities with the affected limb. These benefits have a positive effect on the emotional and psychological states of the patients following injury.

Deep vein thrombosis (DVT) of the lower extremities is a common complication in patients with traumatic fractures, and some studies have shown that the incidence of DVT is significantly higher in patients with lower extremity fractures than in those with fractures at other sites [18]. Seven of the thirty-six patients developed DVT during traction. We believe that patients with a double reverse traction device can achieve significant improvements in lower limb mobility, increased muscle contractions and faster venous blood flow, which may reduce the incidence of deep venous thrombosis. However, slowing of venous blood flow is only one of the factors leading to DVT; the hypercoagulable state caused by injury to the vascular intima and stress in patients after trauma and surgery are also nonnegligible factors that lead to DVT [19, 20].

None of the patients had complications, such as pressure ulcer, pneumonia or secondary neurovascular injury. During the treatment, we found that two patients with distal tibiofibular fractures showed angulation and upturned fracture ends 1 and 2 days after calcaneal traction combined with tibial tuberosity reverse traction. The bone traction pin at the tibial tubercle was replaced at the femoral condyle, and the traction device was reassembled, thus reducing the angulation of the fractured end. In our analysis, the above situation may be due to the fact that the bone traction pin at the calcaneal tuberosity is not in the same horizontal plane as the bone traction pin at the tibial tuberosity. As the pin is pulled upwards at the tibial tubercle, the proximal tibia is blocked by the femur, patella, muscles and soft tissue. Therefore, the actual traction is posterior and superior, thus angling the fracture end anteriorly. We suggest that when treating patients with tibiofibular shaft fractures, bone traction pins should be placed on the supracondylar femur and calcaneal tuberosity to prevent angular displacement of the fracture end, but this method still needs to be further tested in human biomechanical studies.

The limitations of this study include the small sample size, the retrospective nature of the study, and the absence of a control group. Nevertheless, the clinical application of the adjustable PVC tube double counter traction device over the past two years has shown that it can replace the traditional traction method and can be used in the temporary preoperative treatment of lower limb fractures. There are still some shortcomings in the application of this device; for example, the traction force cannot be quantified, the reason why traction is affected the needle position has not been clarified, and the reason why elderly patients with severe osteoporosis show loosening of the bone traction needle after the operation.

## Conclusion

The aim of this study is to introduce a new type of traction device. It is advantageous in that it is light weight, low cost, easy to assemble, promotes immediate movement of the affected limb after assembly, improves patient comfort and can be used with a titanium steel needle for MRI examination under traction. In clinical use, these findings indicate that this may be a safe and appropriate technique, but large cohort studies are needed. It is suitable for the temporary treatment of patients with lower leg fractures prior to surgery, particularly those who, for various reasons, require nonsurgical treatment in the short term. The aim of follow-up research is to improve the device and refine the control group experiments to improve the power of the data and better demonstrate the effectiveness of the device.

### Supplementary Information

**Additional file 1.**

## Data Availability

All datasets analyzed during this study are available from the corresponding author upon reasonable request.
